# Supplementary material for: Mechanochemical synthesis of pillar[5]quinone derived multi-microporous organic polymers for radioactive organic iodide capture and storage
Source: Nat Commun. 2020 Feb 27;11:1086. doi: 10.1038/s41467-020-14892-y (PMC7046611; doi:10.1038/s41467-020-14892-y)
Supplement: Supplementary file 1 — Supplementary Information [file 41467_2020_14892_MOESM1_ESM.pdf]

Supplementary Information

Mechanochemical Synthesis of Pillar[5]quinone-Derived Multi-Microporous Organic Polymers for Radioactive Organic Iodide Capture and Storage

Jie et al.

1. Materials

All commercially available chemical reagents including benzoquinone, 2,5-dichloro-1,4-benzoquinone, 2,5-dibromo-1,4-benzoquinone, iodomethane and triptycene were purchased from Fisher Scientific and used as supplied without further purification. Triptycenehexamine (THA) hexahydrochloride was synthesized according to a procedure described in the literature.[S1] Permethylated pillar[5]arene (MeP5) was synthesized according to a previously reported procedure with a yield over 80%.[S2] Pillar[5]quinone (P5Q) was synthesized by complete oxidation of MeP5 with ammonium cerium nitrate in a mixture of tetrahydrofuran and water.[S3] Perethylated pillar[5]arene (EtP5) was synthesized according to a previously reported procedure with a yield over 60%.[S4] Desolvated EtP5 crystals were obtained by heating at 120 °C in vacuum overnight. Benchmark adsorbents including TED@AC, Ag^+@ZSM-5, Ag^+@13X, Ag^+@MOR, and Ag0@MOR were synthesized according to a literature.[S5]

2. Methods

2.1. Solution NMR. Solution ^1H NMR spectra were recorded at 400 MHz using a JOEL 400 MHz spectrometer.

2.2. Powder X-Ray Diffraction. Powder x-ray diffraction (PXRD) data were collected on a Rigaku Ultimate-IV X-ray diffractometer operating at 40 kV/30 Ma using the Cu Kα line (λ = 1.5418 Å). Data were measured over the range 5–50° in 10°/min steps over 3.5 min.

2.3. Gas Adsorption Analysis. The nitrogen adsorption and desorption isotherms were measured at 77 K under a Gemini 2360 surface area analyzer. The CO_2 adsorption and desorption isotherms were measured at 273 K and 298 K by Autosorb-1-C Quantachrome analyzer. Samples were degassed under dynamic vacuum for 12 h at 150 °C prior to each measurement.

2.4. Vapor Sorption Measurement. CH_3I vapor sorption isotherms were measured *via* Micromeritics 3Flex. Samples were degassed under dynamic vacuum for 12 h at 150 °C prior to each measurement.

2.5. Thermogravimetric Analysis. Thermogravimetric analysis (TGA) was carried out using a Q5000IR analyzer (TA instruments) with an automated vertical overhead thermobalance. The samples were heated at the rate of 10 °C/min using N_2 as the protective gas.

2.6. The Fourier Transform Infrared (FT-IR) Spectroscopy. The FT-IR spectra were recorded from KBr pellets containing ca. 1 mg of the compound in the range 4000–400 cm^{-1} on a Perkin–Elmer one FT-IR spectrophotometer.

2.7. ^{13}C Solid-State NMR. The ^{13}C solid-state NMR spectra were acquired at room temperature on a using a solid-state Varian INOVA 400 MHz. spectrometer. The experiments were performed under magic angle spinning (MAS) at 6.1 kHz with a recycle delay of 3.5 s and using cross-polarization (CP) with a ^{13}C radio-frequency (rf) field amplitude of 41 kHz ramped to obtain maximum signal at a ^{1}H rf field of approximately 65 kHz and with an optimized contact pulse of 1.5 ms.

2.8. High-Resolution Transmission Electron Microscopy (HR-TEM) and Energy-Dispersive X-ray Spectroscopy (EDS). High-resolution transmission electron microscopy (HR-TEM) and HR-TEM-EDS investigations were performed on a JEM 2100F instrument.

2.9. Scanning Electron Microscopy. Scanning electron microscopy (SEM) investigations were carried out on a HITACHI SU-8010 instrument at an electron acceleration voltage of 15 kV.

2.10. Single Crystal Growth. Single crystals of CH_3I loaded EtP5 were grown by slow evaporation method: 2.5 mg of dry EtP5 powders were dissolved in 2 mL of CH_3I in a small vial where. The resultant transparent solution was allowed to evaporate slowly to give nice colourless crystals in 2 days.

2.11. Single Crystal X-ray Diffraction

Single crystal X-ray data sets were measured on a Rigaku MicroMax-007 HF rotating anode diffractometer (Mo-Kα radiation, λ = 0.71073 Å, Kappa 4-circle goniometer, Rigaku Saturn724+ detector). Supplementary CIFs, which include structure factors, are available free of charge from the Cambridge Crystallographic Data Centre (CCDC) via www.ccdc.cam.ac.uk/data_request/cif.

2.12. X-Ray Photoelectron Spectroscopy. X-ray photoelectron spectroscopy (XPS) was performed on a PHI 3056 spectrometer equipped with an Al anode source operated at 15 kV and an applied power of 350 W and a pass energy of 93.5 eV. Samples were mounted on foil since the C1*s* binding energy was used to calibrate the binding energy shifts of the sample (C1*s* = 284.8 eV).

2.13. Elemental Analyses. Elemental analyses were performed on a Perkin-Elmer 2400 element analyzer.

2.14. Electrospray Ionization Mass Spectrometry (ESI MS). Electrospray ionization mass spectrometry (ESI MS) were obtained on a Bruker Esquire 3000 plus mass spectrometer (Bruker-Franzen Analytik GmbH Bremen, Germany) equipped with an ESI interface and an ion trap analyzer.**.** Elemental analyses were performed on a Perkin-Elmer 2400 element analyzer.

3. Crystallography Data

Supplementary Table 1. Experimental single-crystal X-ray data for CH_3I-loaded EtP5 structure.

Formula	EtP5·1.75(CH_3I)
Crystallisation Solvent	CH_3I
Collection Temperature	170 K
Formula	$C_{56.75}H_{74.25}I_{1.75}O_{10}$
Mr	1138.48
Crystal Size [mm]	0.05 × 0.03 × 0.02
Crystal System	Orthorhombic
Space Group	*Pbcn*
a [Å]	42.221(3)
b [Å]	15.9481(10)
c [Å]	16.9721(11)
α [°]	
β [°]	
γ [°]	
V [Å^3]	11427.9(13)
Z	8
D_{calcd} [g cm^{-3}]	1.323
μ[mm^{-1}]	5.484
F(000)	4700
2θ range [°]	6.86 – 110.94
Reflections collected	105644
Independent reflections, R_{int}	11027, 0.0877
Obs. Data [$I > 2\sigma(I)$]	5042
Data /restraints / parameters	11027/ 38 / 653
Final R_1 values ($I > 2\sigma(I)$)	0.1219
Final R_1 values (all data)	0.2079
Final $wR(F_2)$ values (all data)	0.3340
Goodness-of-fit on F^2	0.985
Largest difference peak and hole [e.A^{-3}]	2.438 / -1.073
CCDC	1948606

3. Synthetic Details

3.1 Synthesis of Triptycenehexaamine (THA) Hexahydrochloride.

Two-step synthesis of air-stable triptycenehexaamine hexachloride **4**.[x]

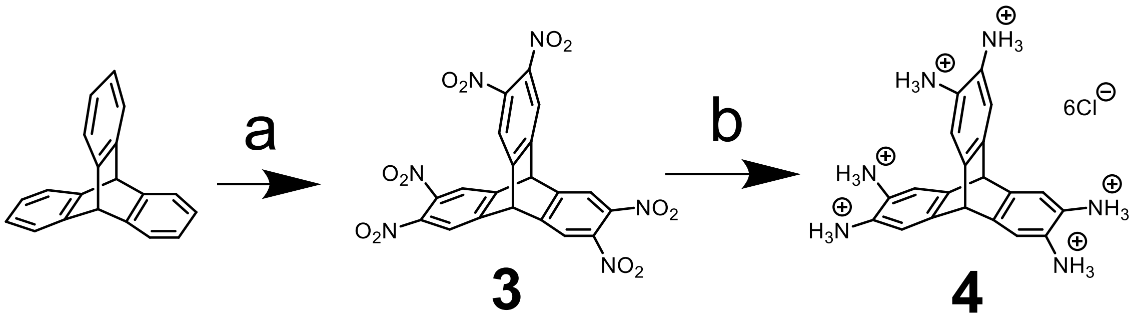

[x]Key: (a) fuming nitric acid, 85 °C, 4 h; (b) $SnCl_2{\cdot}2H_2O$, EtOH, HCl_{aq} (conc), reflux, 17 h.

Triptycene (5.15 g, 20.2 mmol) was suspended in fuming nitric acid (150 mL, 100%) and heated to 85 °C for 4 h. The reaction mixture was cooled to room temperature, poured into water (1 L), and stirred for 1 h. The pale yellow (slightly pink) precipitate was collected by suction filtration, washed with water, and dried in air to give approximately 11 g of crude product. Recrystallization from hot DMF (reflux temperature) gives after cooling to room temperature 3 as yellow crystals (1.85 g, 18%). A suspension of **3** (1.25 g, 2.4 mmol) and tin(II) chloride dihydrate (18 g, 79 mmol) in ethanol (140 mL) and concentrated hydrochloric acid (60 mL) was refluxed for 24 h. The reaction mixture was cooled to room temperature and the white precipitate collected by filtration, washed with concentrated hydrochloric acid (3 × 15 mL), and dried in vacuum to give **4** as pale yellow solids (1.70 g (quant): mp 288 °C; ^1H NMR (400 MHz, DMSO-d_6) δ 7.04 (s, 6H), 5.32 (s, 2H); ^{13}C NMR (126 MHz, DMSO-d_6) δ 140.56, 126.04, 116.42, 50.45. The ESI MS spectrum is shown in Supplementary Figure 3: *m*/*z* 326.88 [**4**$\cdot 5H_2O + 2H]^{2+}$.

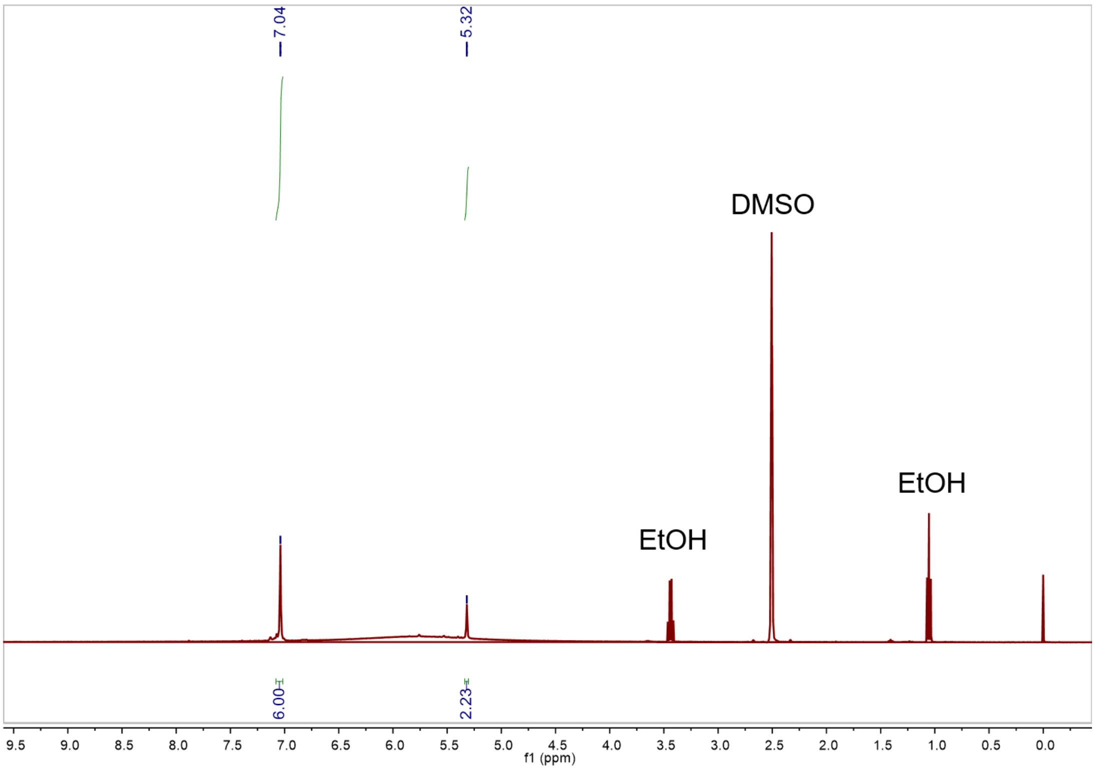

Supplementary Figure 1. ^{1}H NMR spectrum (400 MHz, DMSO-d_6, 293 K) of triptycenehexaamine (THA) hexahydrochloride **4**.

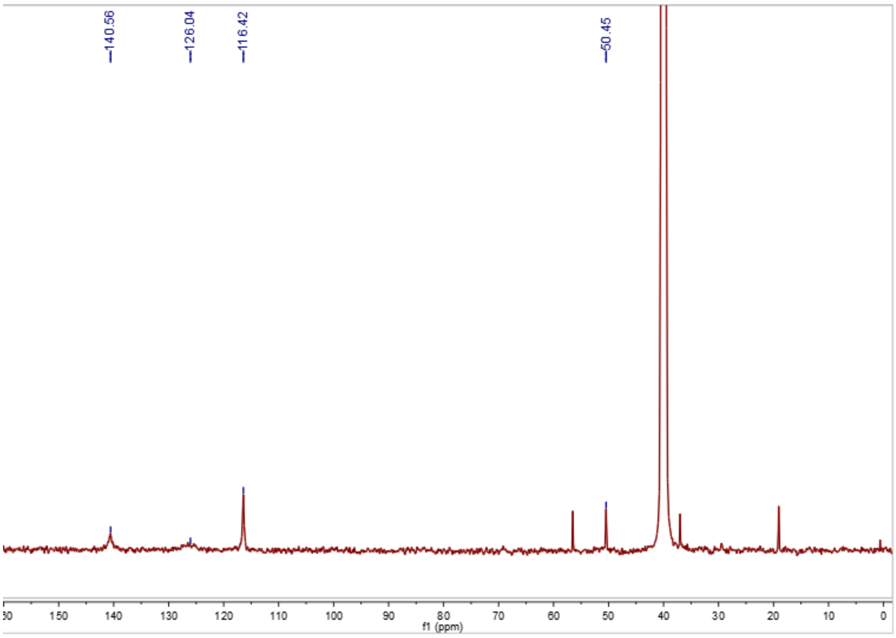

Supplementary Figure 2. ^{13}C NMR spectrum (125 MHz, DMSO-d_6, 293 K) of triptycenehexaamine (THA) hexahydrochloride **4**.

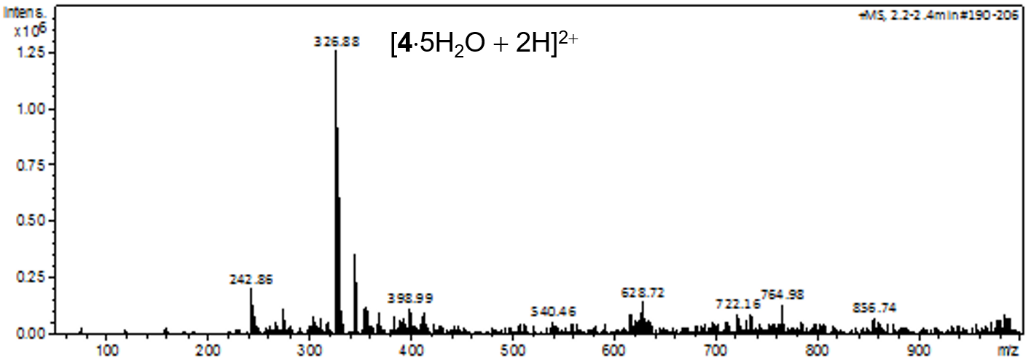

Supplementary Figure 3. ESI MS spectrum of triptycenehexaamine (THA) hexahydrochloride **4**.

3.2 Synthesis of Model Compound.

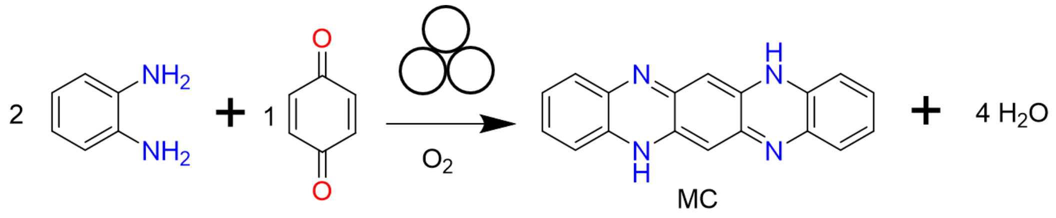

Benzoquinone (0.108 g; 1 mmol) and *o*-phenylenediamine (0.216 g; 2 mmol) were added into a 15 mL stainless steel grinding jar (33 mm diameter) with 3 steel balls. The mixture was then ground for 30 min in a Retsch MM400 grinder mill operating at 30 Hz. The crude product was washed with saturated $NaHCO_3$ and then extracted with CH_2Cl_2 (3 × 30 mL), and the organic extracts were combined, dried (Na_2SO_4), concentrated in vacuo, and filtered. The residue was purified by flash column chromatography (silica gel, *n*-hexane–ethyl acetate, 90:10 to 40:60) to give the model compound MC with over 95% yield. ^{1}H NMR (400 MHz, DMSO-d_6) δ 7.87-7.89 (m, 4H), 7.53-7.55 (m, 4H), 6.89 (s, 2H), 6.27 (s, 2H). ^{13}C NMR (125 MHz, DMSO-d_6) δ 146.40, 143.73, 141.20, 128.53, 128.07. The ESI MS: m/z 282.93 $[MC - H]^{-}$.

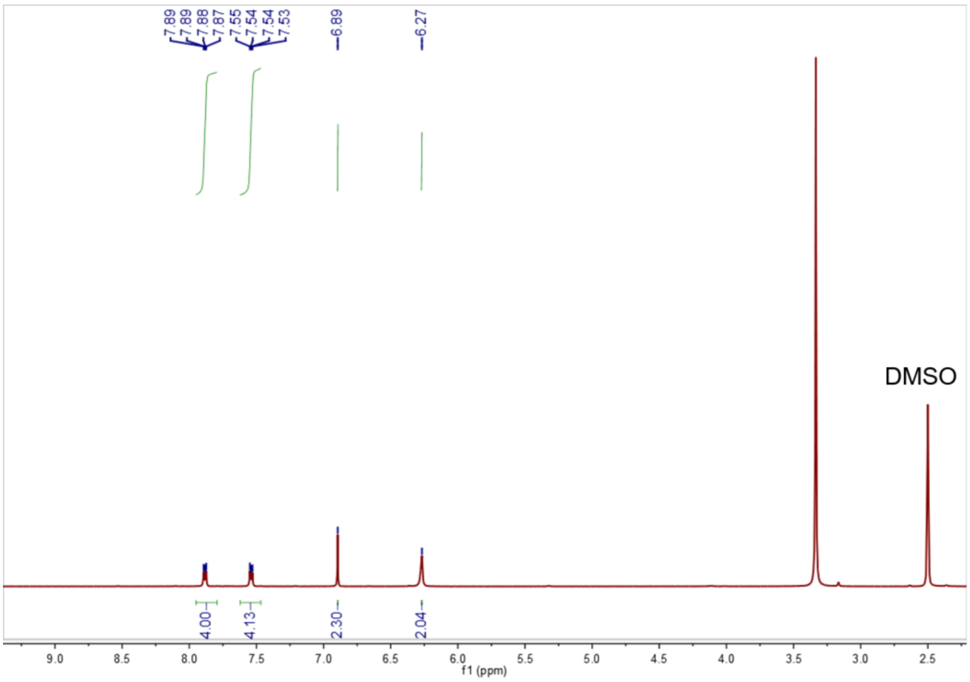

Supplementary Figure 4. ^{1}H NMR spectrum (400 MHz, DMSO-d_6, 293 K) of model compound MC.

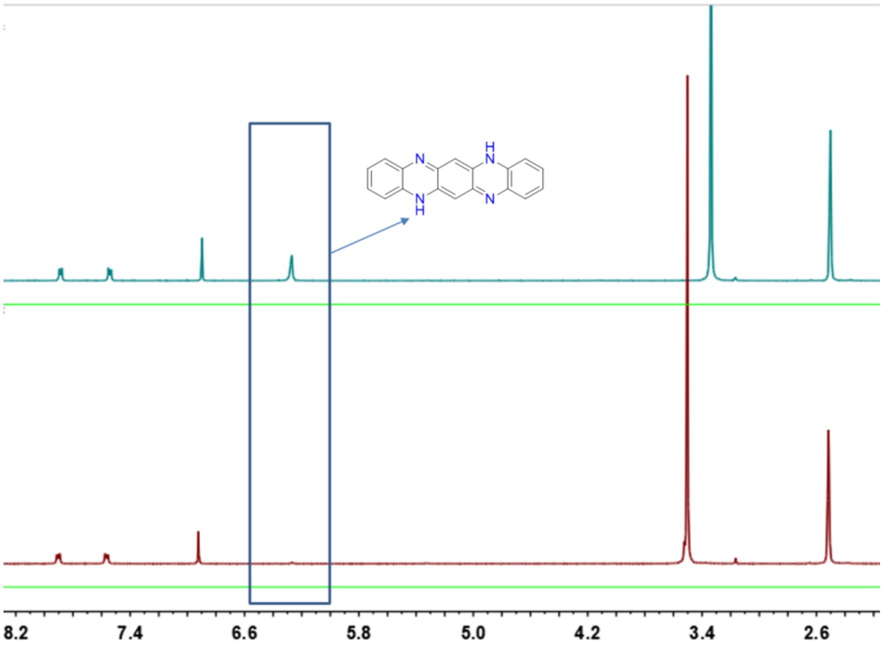

Supplementary Figure 5. ^{1}H NMR spectrum (400 MHz, DMSO-d_6, 293 K): model compound MC (top); model compound MC in the presence of a drop of D_2O (bottom).

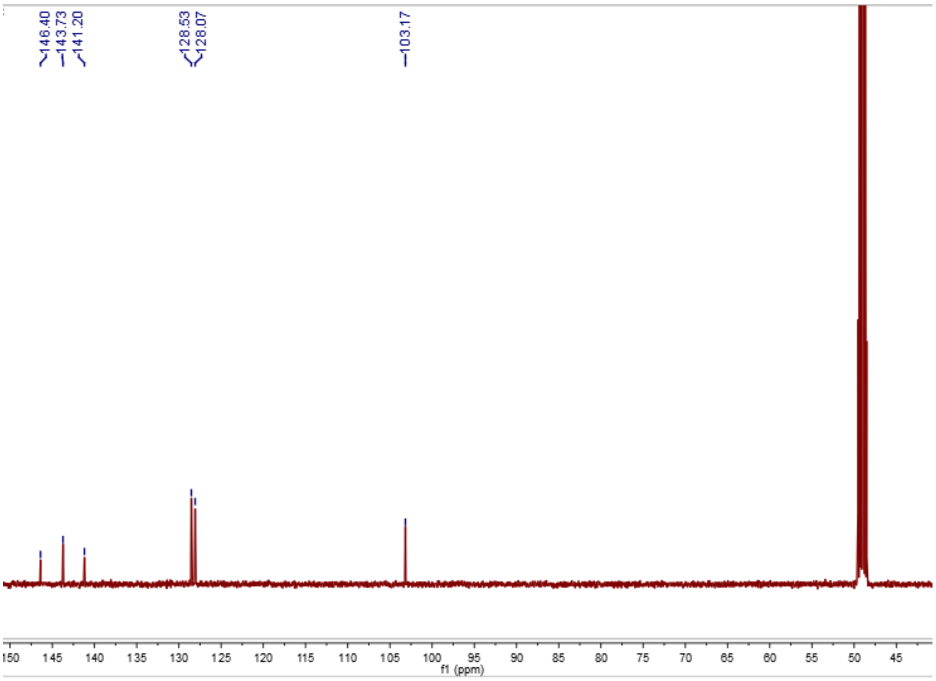

Supplementary Figure 6. ^{13}C NMR spectrum (125 MHz, DMSO-d_6, 293 K) of model compound MC.

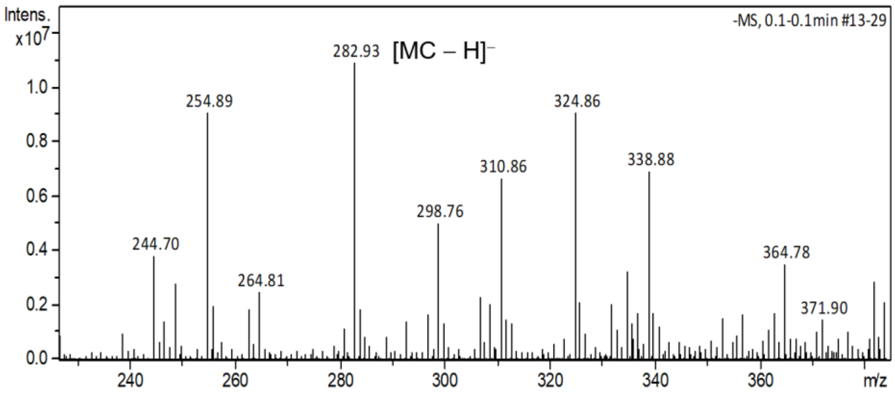

Supplementary Figure 7. ESI MS spectrum of model compound MC.

3.3 Synthesis of MHPs

3.3.1 Synthesis of MHP

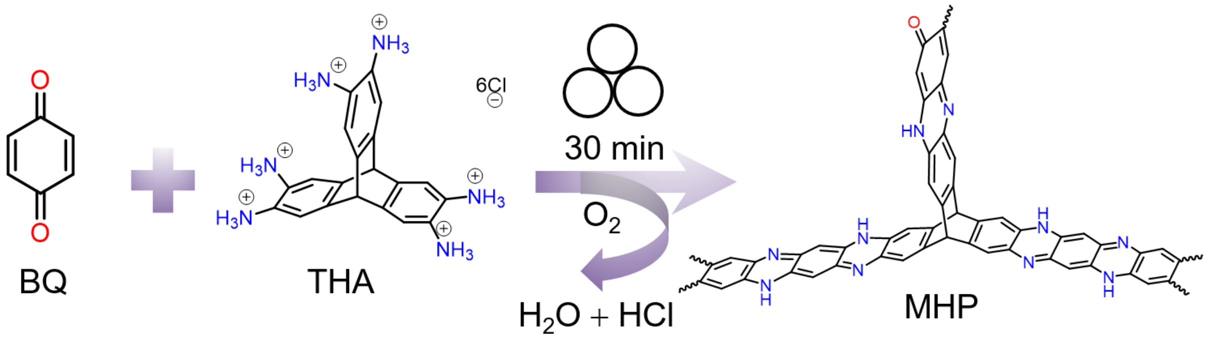

Benzoquinone (0.108 g; 1 mmol) and triptycenehexaamine hexahydrochloride (0.374 g; 0.67 mmol) were added into a 15 mL stainless steel grinding jar (33 mm diameter) with 3 steel balls. The mixture was then ground for 30 min in a Retsch MM400 grinder mill operating at 30 Hz. The solids were washed with saturated $NaHCO_3$ solution for 2 times and methanol for 4 times, which were dried at 100 °C for 12 hours to give dark brown powders (0.148 g).

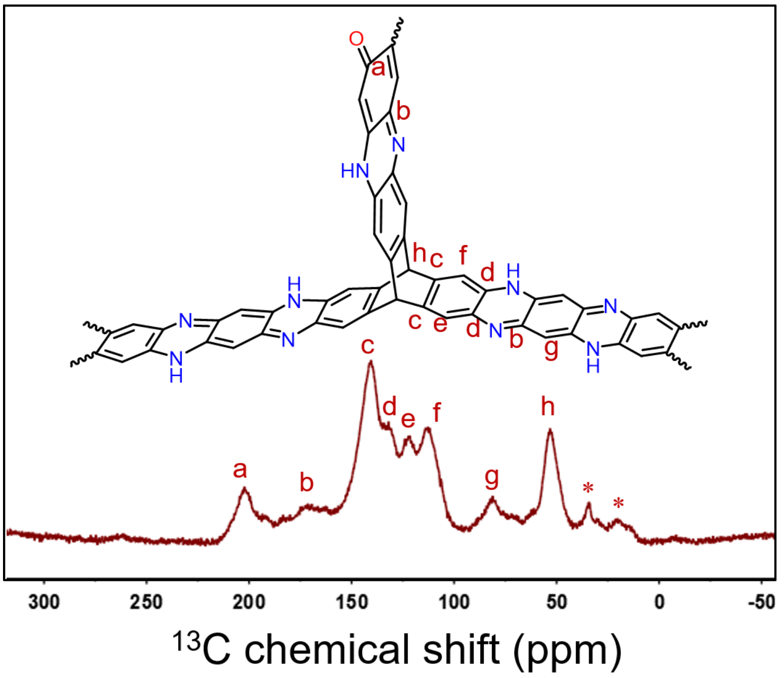

Supplementary Figure 8. ^{13}C CP-MAS NMR spectrum of MHP.

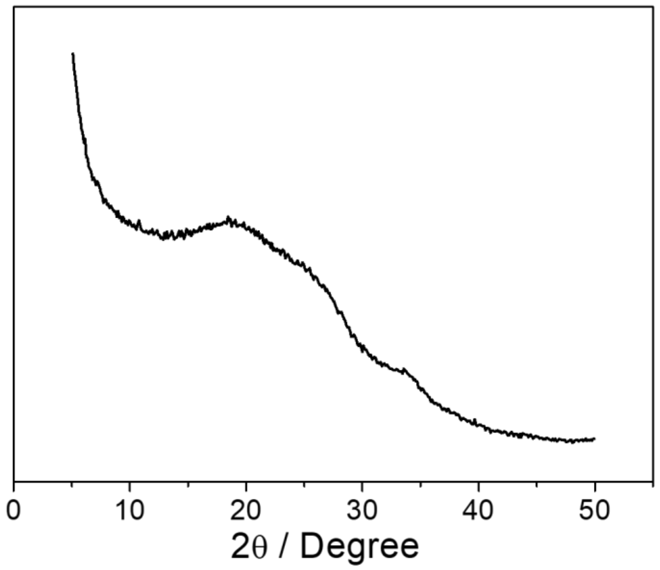

Supplementary Figure 9. PXRD pattern of MHP.

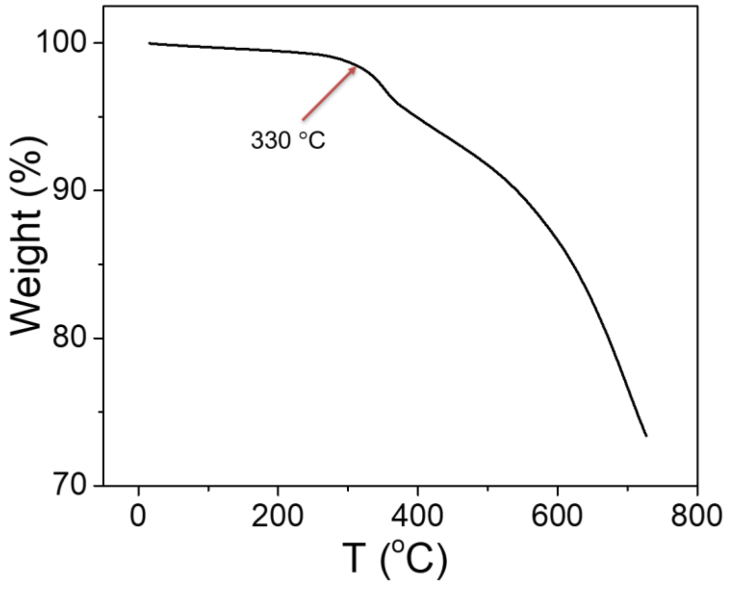

Supplementary Figure 10. TGA curve of MHP. Almost no weight loss occurred below 330 °C, indicating its thermostability below 330 °C.

3.3.2 Synthesis of MHP-Cl

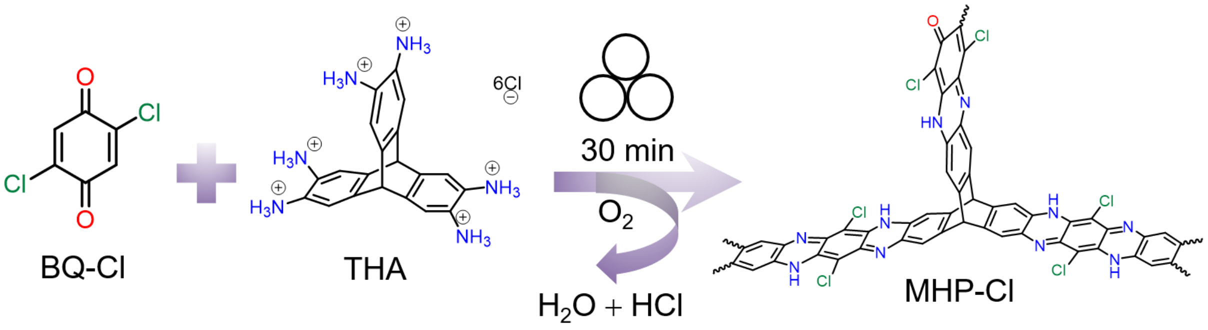

The synthesis of MHP-Cl is pretty similar to that of MHP. In particular, 2,5-dichloro-1,4-benzoquinone (0.176 g; 1 mmol) and triptycenehexaamine hexahydrochloride (0.374 g; 0.67 mmol) were added into a 15 mL stainless steel grinding jar (33 mm diameter) with 3 steel balls. The mixture was then ground for 30 min in a Retsch MM400 grinder mill operating at 30 Hz. The solids were washed with saturated $NaHCO_3$ solution for 2 times and methanol for 4 times, which were dried at 100 °C for 12 hours to give dark brown powders (0.218 g).

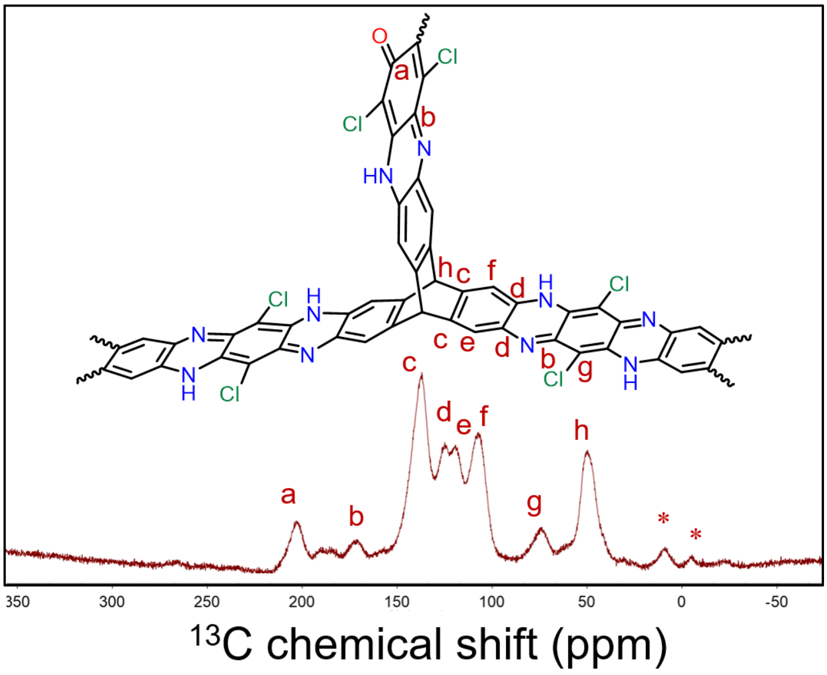

Supplementary Figure 11. ^{13}C CP-MAS NMR spectrum of MHP.

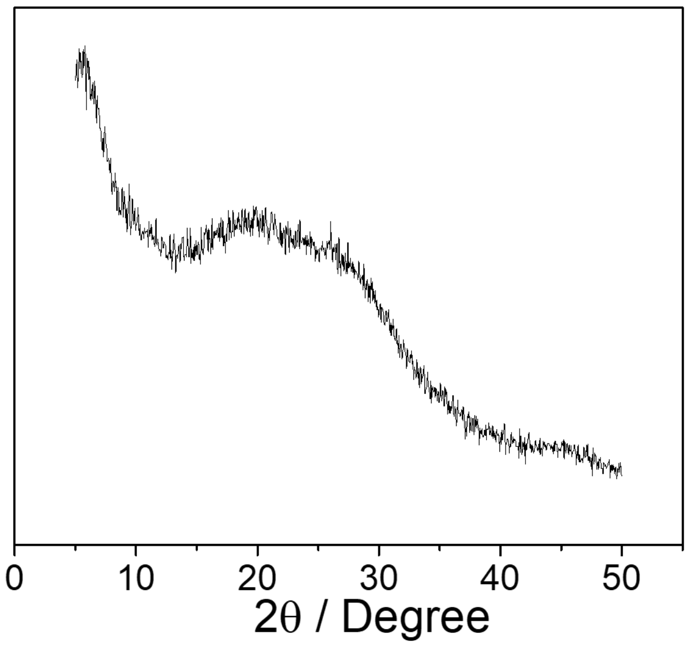

Supplementary Figure 12. PXRD pattern of MHP-Cl.

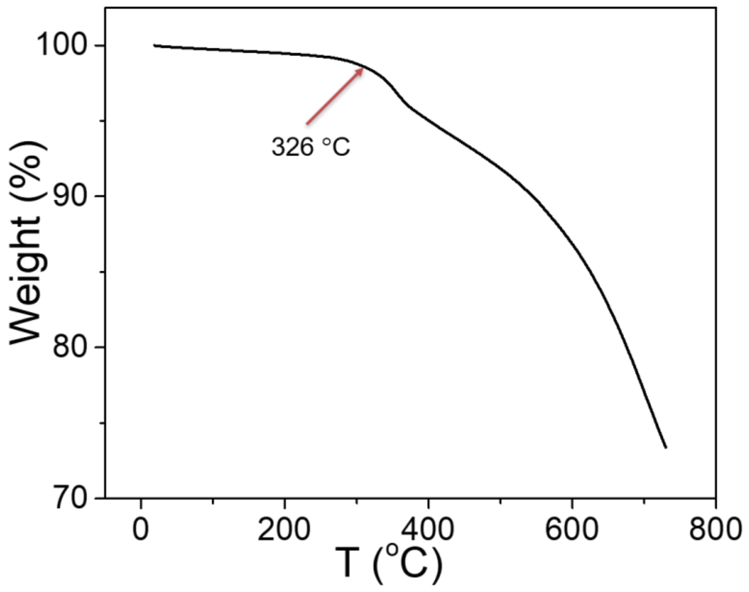

Supplementary Figure 13. TGA curve of MHP-Cl. Almost no weight loss occurred below 326 °C, indicating its thermostability below 326 °C.

3.3.3 Synthesis of MHP-Br

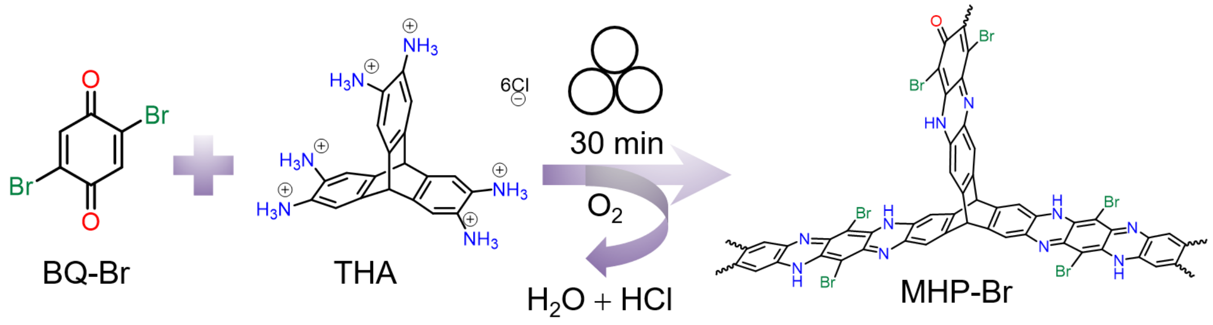

The synthesis of MHP-Br is pretty similar to that of MHP. In particular, 2,5-dibromo-1,4-benzoquinone (0.265 g; 1 mmol) and triptycenehexaamine hexahydrochloride (0.374 g; 0.67 mmol) were added into a 15 mL stainless steel grinding jar (33 mm diameter) with 3 steel balls. The mixture was then ground for 30 min in a Retsch MM400 grinder mill operating at 30 Hz. The solids were washed with saturated $NaHCO_3$ solution for 2 times and methanol for 4 times, which were dried at 100 °C for 12 hours to give dark brown powders (0.276 g).

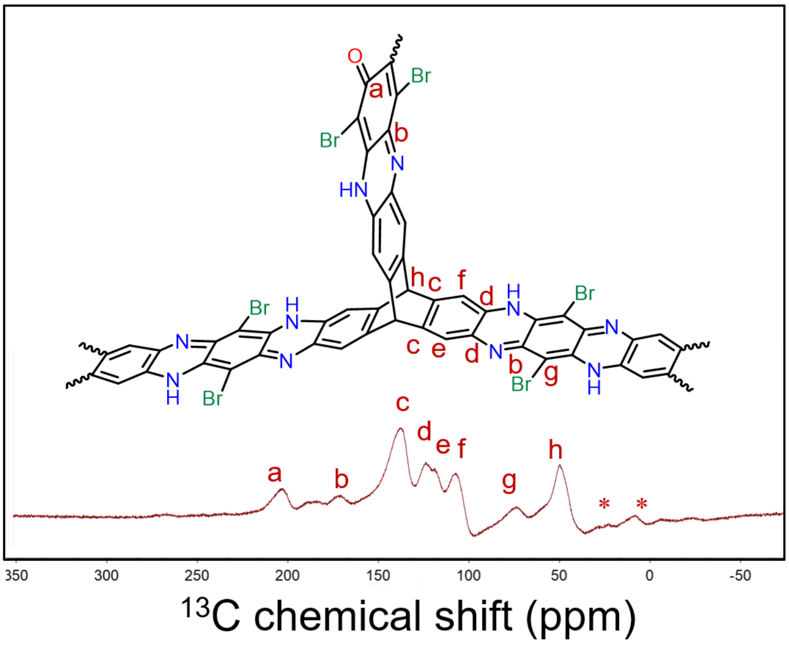

Supplementary Figure 14. ^{13}C CP-MAS NMR spectrum of MHP-Br.

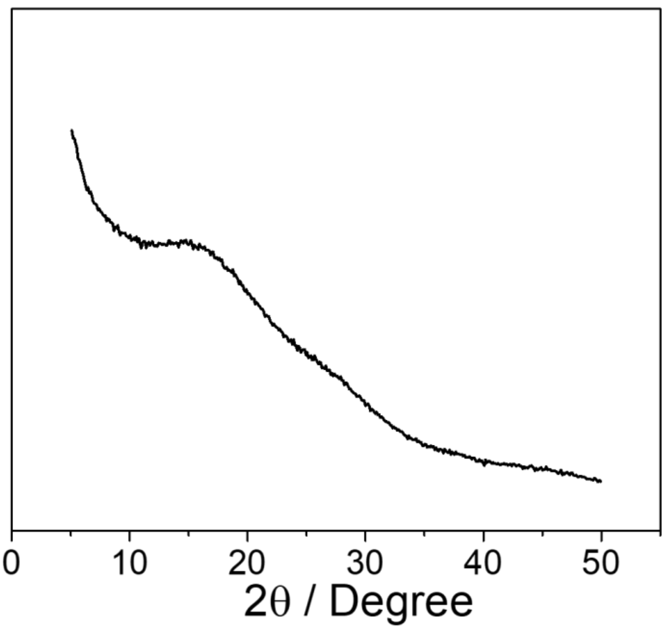

Supplementary Figure 15. PXRD pattern of MHP-Br.

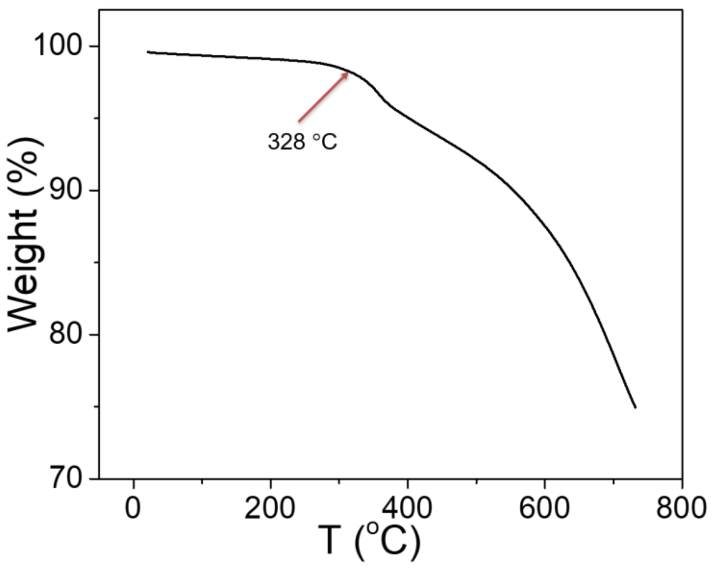

Supplementary Figure 16. TGA curve of MHP-Br. Almost no weight loss occurred below 328 °C, indicating its thermostability below 328 °C.

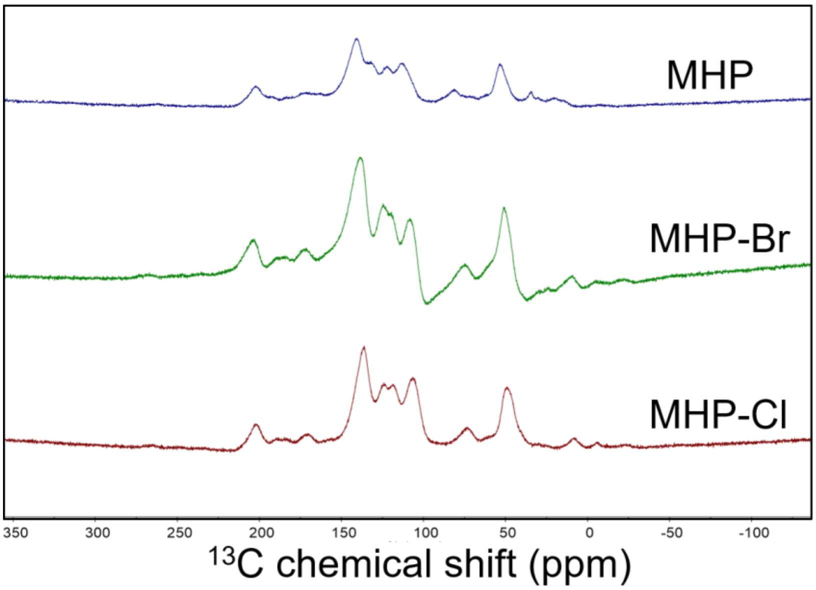

Supplementary Figure 17. Comparison of the ^{13}C CP-MAS NMR spectra of MHP, MHP-Cl, and MHP-Br.

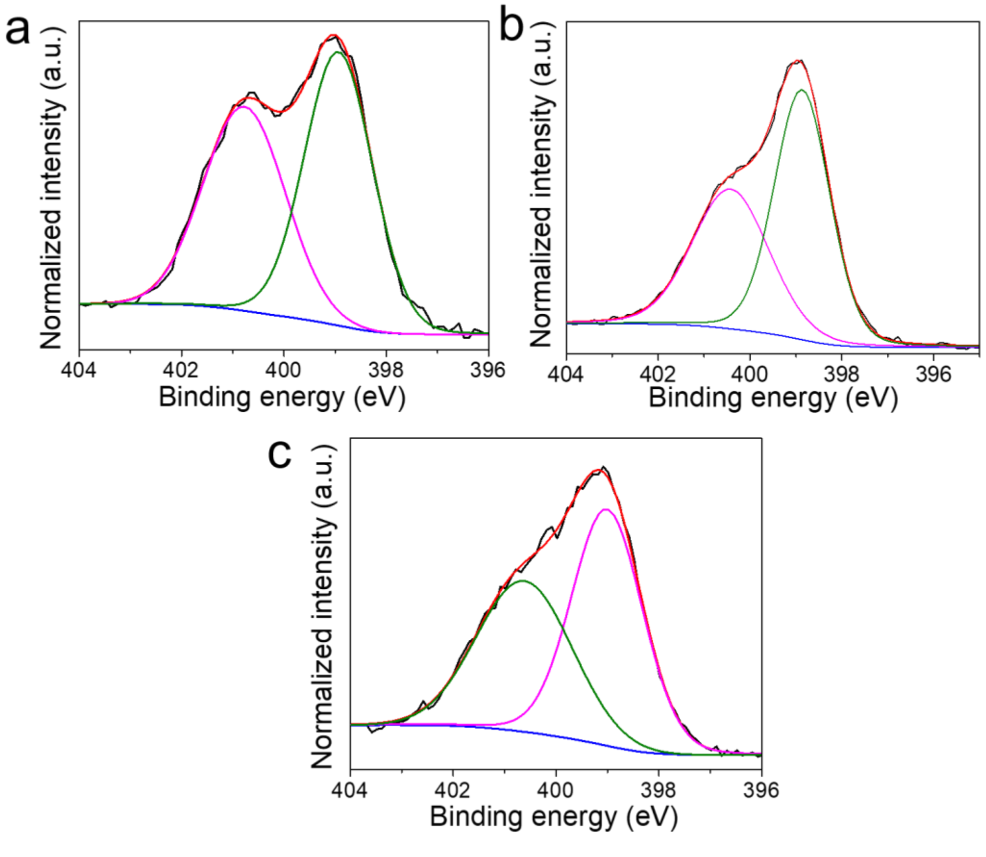

Supplementary Figure 18. N 1*s* XPS spectra of (a) MHP, (b) MHP-Cl, and (c) MHP-Br.

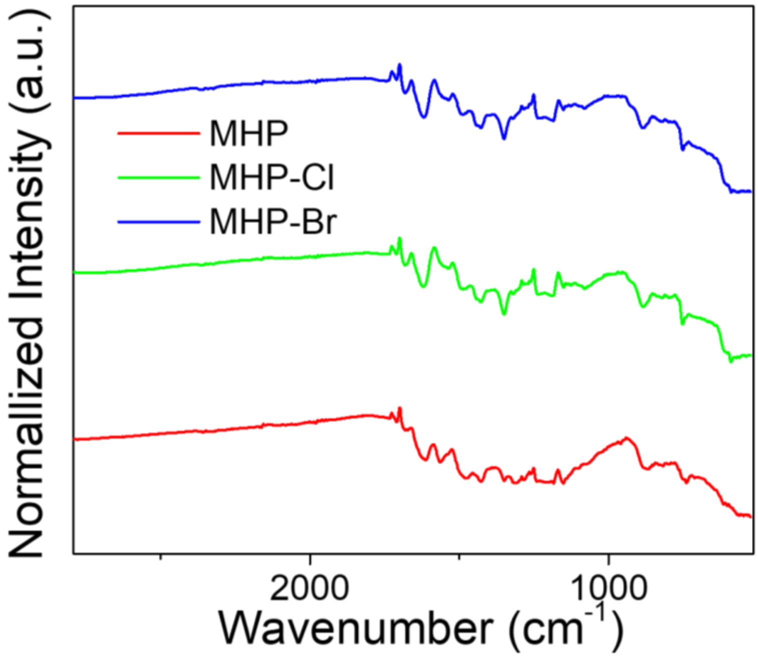

Supplementary Figure 19. FT-IR spectra of MHP, MHP-Cl, and MHP-Br.

3.3.4 Synthesis of MHP-P5Q

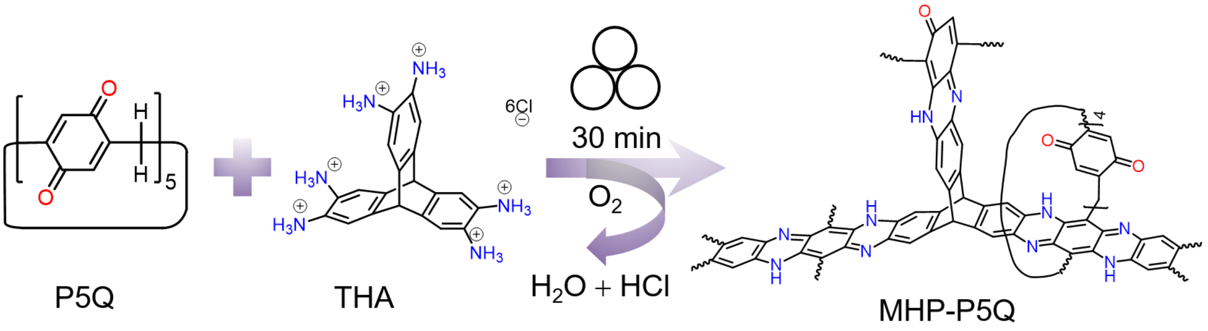

The synthesis of MHP-P5Q is pretty similar to that of MHP. In particular, P5Q (0.150 g; 0.25 mmol) and triptycenehexaamine hexahydrochloride (0.468 g; 0.838 mmol) were added into a 15 mL stainless steel grinding jar (33 mm diameter) with 3 steel balls. The mixture was then ground for 30 min in a Retsch MM400 grinder mill operating at 30 Hz. The solids were washed with saturated $NaHCO_3$ solution for 2 times and methanol for 4 times, which were dried at 100 °C for 12 hours to give dark brown powders (0.204 g).

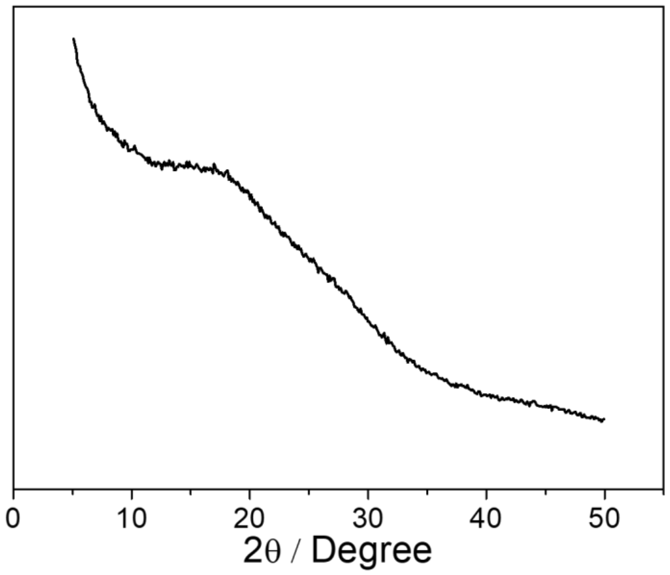

Supplementary Figure 20. PXRD pattern of MHP-P5Q.

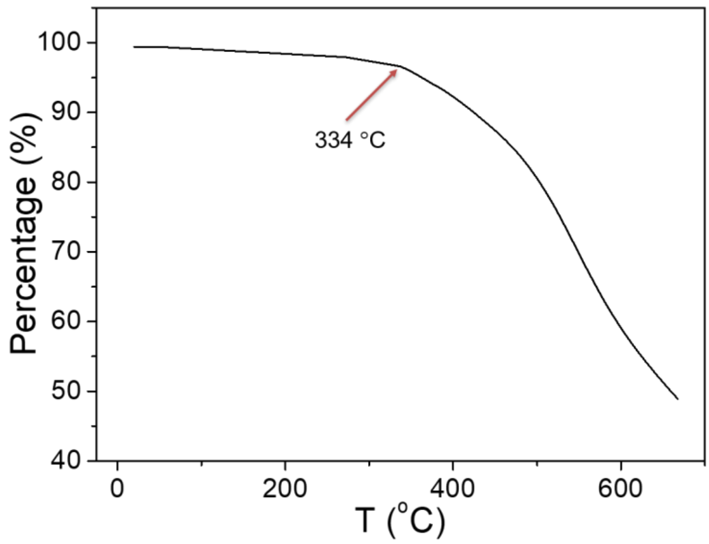

Supplementary Figure 21. TGA curve of MHP-P5Q. Almost no weight loss occurred below 334 °C, indicating its thermostability below 334 °C.

3.4 Synthesis of P5Q-TAB and P5Q-DAB

3.4.1 Synthesis of P5Q-TAB

P5Q (0.150 g; 0.25 mmol) and 1,2,4,5-tetraaminobenzene tetrahydrochloride (0.355 g; 1.25 mmol) were added into a 15 mL stainless steel grinding jar (33 mm diameter) with 3 steel balls. The mixture was then ground for 30 min in a Retsch MM400 grinder mill operating at 30 Hz. The solids were washed with saturated $NaHCO_3$ solution for 2 times and methanol for 4 times, which were dried at 100 °C for 12 hours to give dark brown powders (0.204 g).

3.4.2 Synthesis of P5Q-DAB

P5Q (0.150 g; 0.25 mmol) and 3,3'-diaminobenzidine (0.268 g; 1.25 mmol) were added into a 15 mL stainless steel grinding jar (33 mm diameter) with 3 steel balls. The mixture was then ground for 30 min in a Retsch MM400 grinder mill operating at 30 Hz. The solids were washed with saturated $NaHCO_3$ solution for 2 times and methanol for 4 times, which were dried at 100 °C for 12 hours to give dark brown powders (0.204 g).

4. Gas Sorption Data

Supplementary Table 2. Porosity Parameters of MHPs.

MHPs	S_{BET} (m^2/g)	V_{total} (cm^3/g)	V_{micro} (cm^3/g)	V_{micro}/V_{total}
MHP	320	0.16	0.11	0.69
MHP-Cl	289	0.14	0.105	0.75
MHP-Br	208	0.11	0.08	0.73
MHP-P5Q	296	0.13	0.10	0.77

Supplementary Table 3. BET surface area of MHP-P5Q in different conditions.

	Boiling water	Water steam	1 M NaOH	0.1 mM HCl	1 M HCl
BET surface area (m^2/g)	302	293	295	226	7

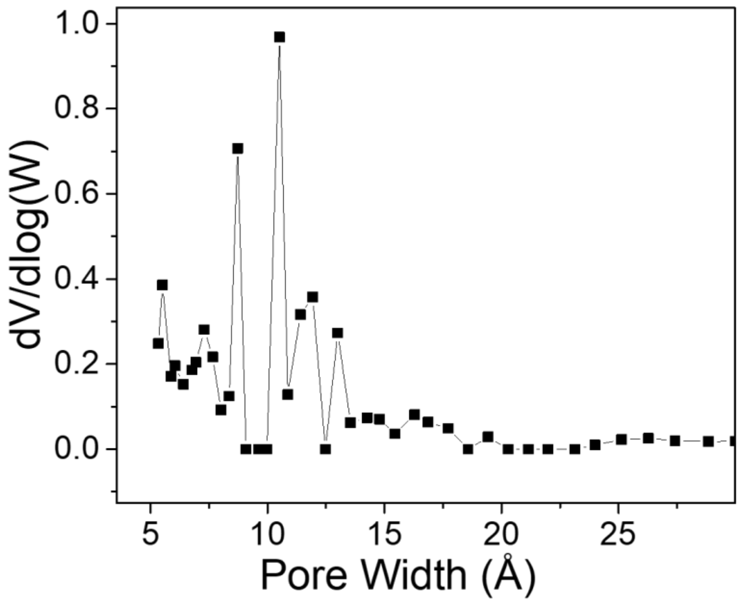

Supplementary Figure 22. Pore size distribution of MHP calculated from the NLDFT approximation.

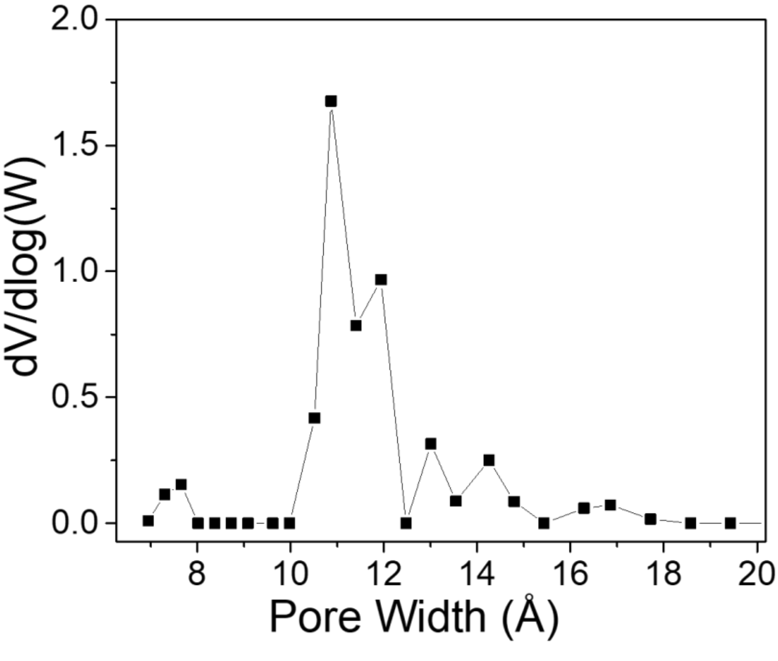

Supplementary Figure 23. Pore size distribution of MHP-Cl calculated from the NLDFT approximation.

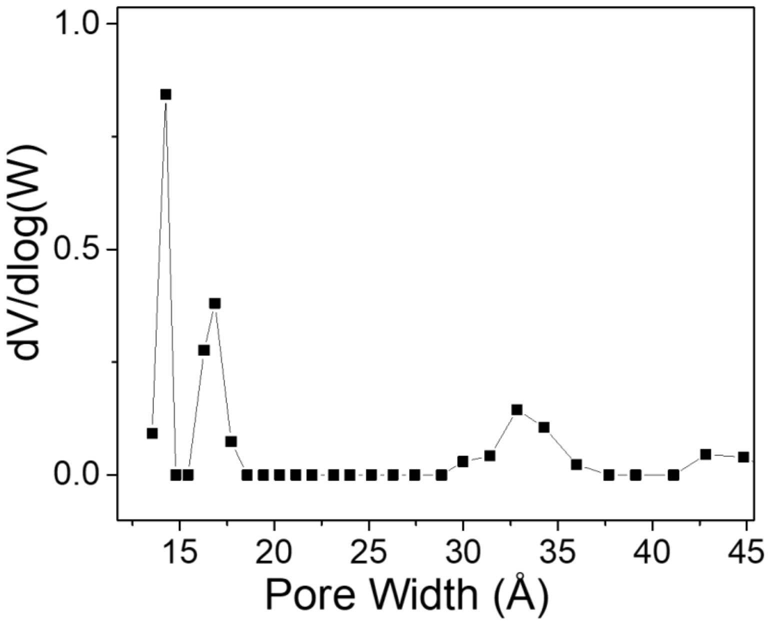

Supplementary Figure 24. Pore size distribution of MHP-Br calculated from the NLDFT approximation.

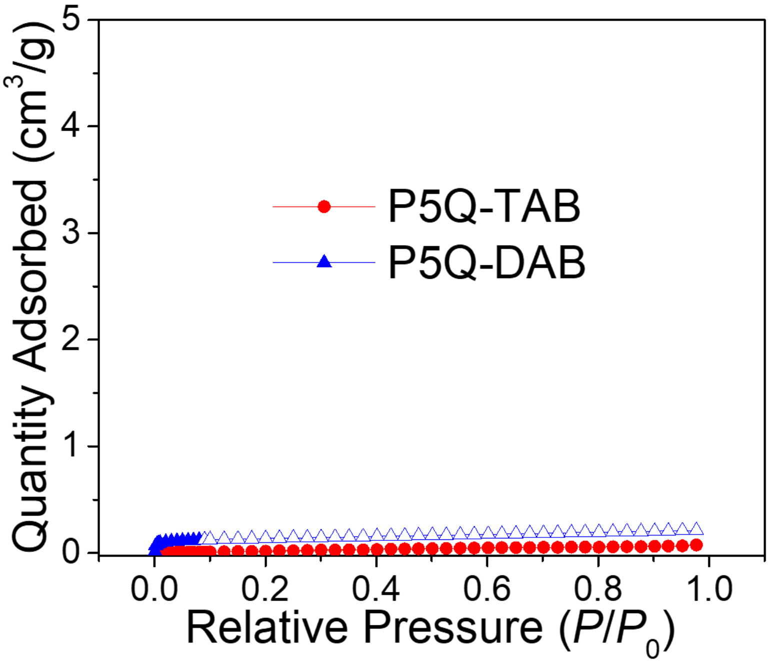

Supplementary Figure 25. N_2 adsorption and desorption isotherms of P5Q-TAB and P5Q-DAB.

5. CH_3I Vapor Capture Experiment

For CH_3I vapor capture experiment from the air, an open 5 mL vial containing 20 mg of MHPs or EtP5 was placed in a sealed 20 mL vial containing 2 mL of CH_3I. Time-dependent CH_3I uptake in the adsorbents was obtained by measuring the weight of the adsorbents at room temperature or at 100 °C. For the sSupplementary Table torage of CH_3I in these adsorbents, the adsorbents after capturing of CH_3I from air were exposed to atmosphere for 30 days at room temperature. The reserved CH_3I uptake in the adsorbents was obtained by measuring the weight of the adsorbents.

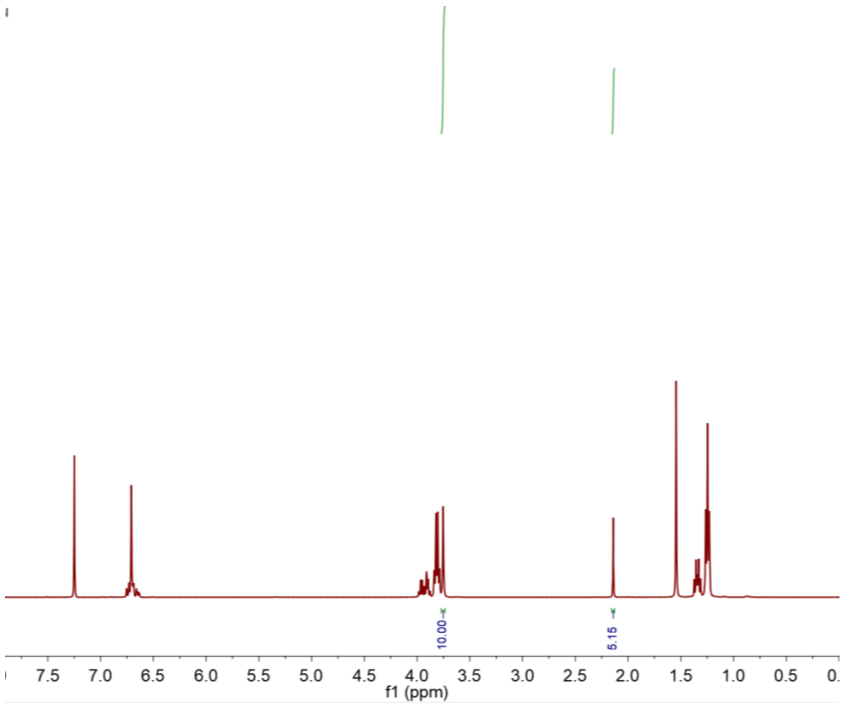

Supplementary Figure 26. ^1H NMR spectrum (400 MHz, $CDCl_3$, 293 K) of EtP5 after adsorption of CH_3I vapor.

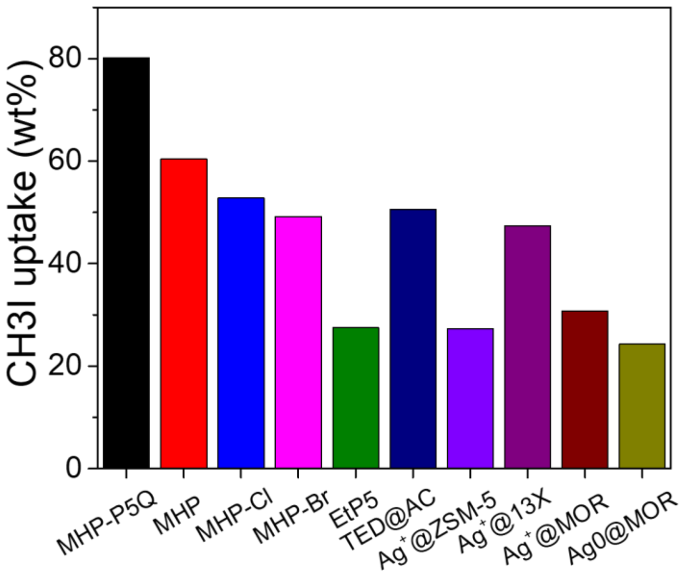

Supplementary Figure 27. Comparing the saturated CH_3I uptake in MHPs and selected benchmark sorbent materials at room temperature.

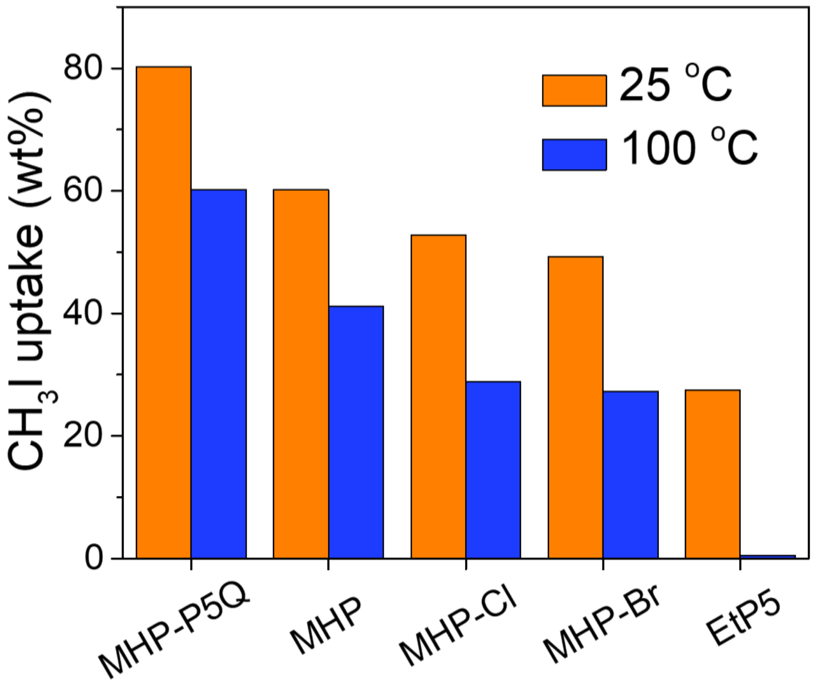

Supplementary Figure 28. Comparison of CH_3I uptake in MHPs and EtP5 at 25 °C and 100 °C.

6. Mechanistic Studies on CH_3I Capture

6.1 Characterizations of CH_3I@MHP-P5Q and CH_3I@MHP

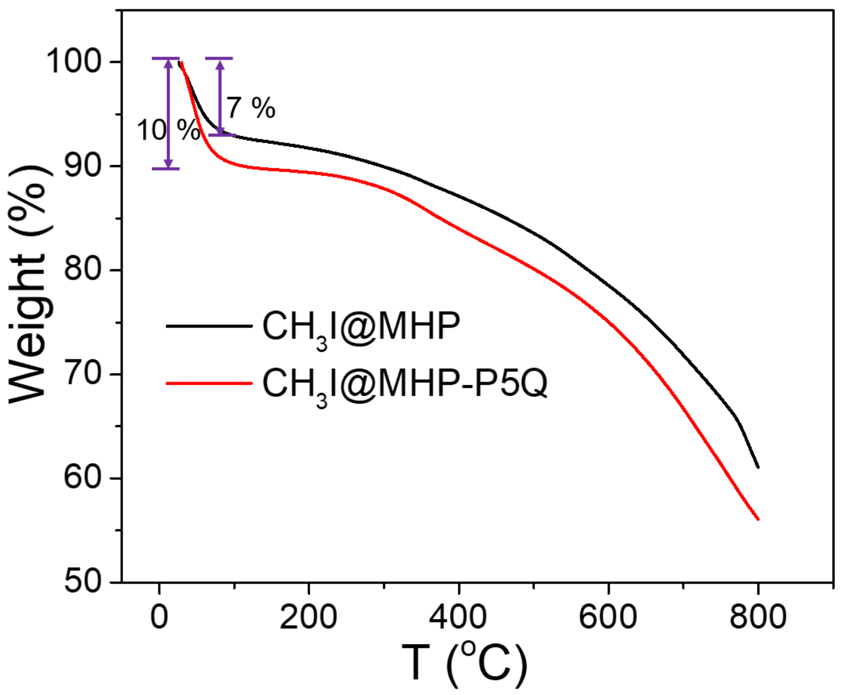

Supplementary Figure 29. TGA curves of CH_3I@MHP and CH_3I@MHP-P5Q. The weight loss below 100 °C are 7 wt% and 10 wt% for CH_3I@MHP and CH_3I@MHP-P5Q, respectively, indicating that only a small fraction of the captured CH_3I was released at the temperature higher than its boiling point

6.2 Synthesis and Characterizations of MC-CH_3I

Model compound MC (0.025 g) was dissolved in methanol (5 mL) in a small vial, where 1 mL CH_3I was charged. The resulting solution was stirred at room temperature for 1 hour. After this, the solvent was removed through rotary evaporation to give the product MC-CH_3I as dark solids.

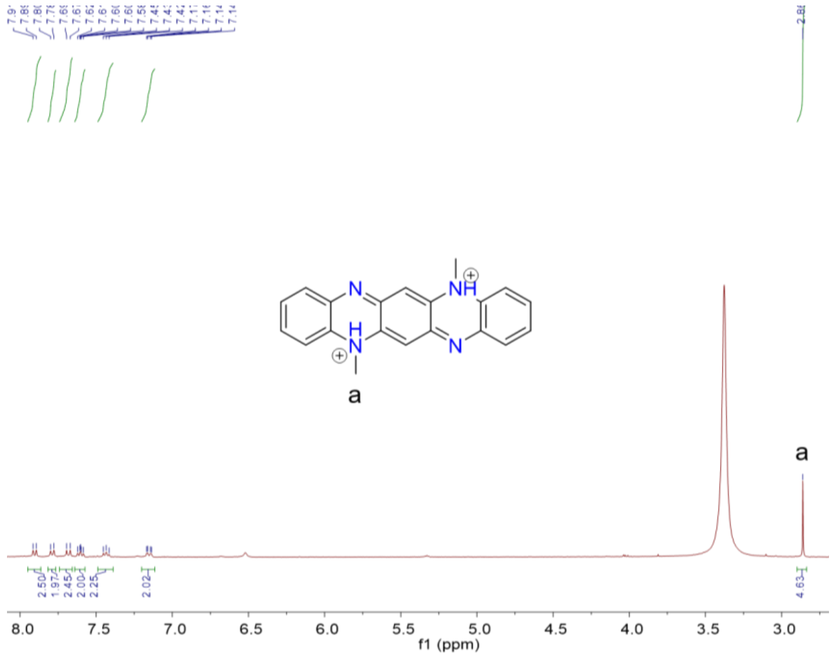

Supplementary Figure 30. ^1H NMR spectrum (400 MHz, $CDCl_3$, 293 K) of MC-CH_3I.

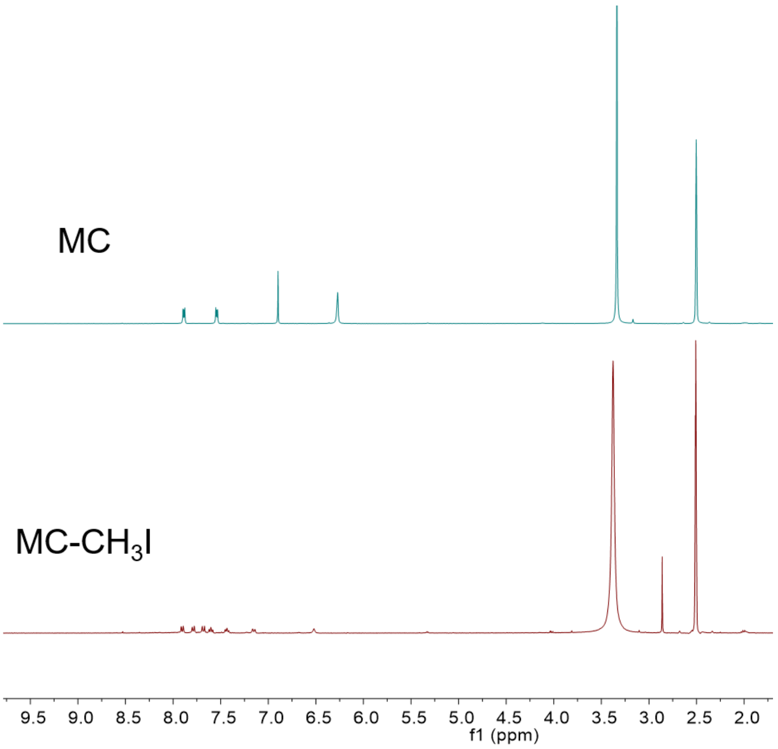

Supplementary Figure 31. Comparison of the ^1H NMR spectrum (400 MHz, $CDCl_3$, 293 K) of MC and MC-CH_3I.

6.3 Synthesis and Characterizations of MHP-P5Q-CH_3I and MHP-CH_3I

MHP-P5Q or MHP (0.020 g) was charged into in a small vial containing 5 mL methanol and 1 mL CH_3I. The resulting suspension was stirred at room temperature for 2 hours. After this, the solid was filtered and dried at 120 °C in vacuum overnight to give the product MHP-P5Q-CH_3I or MHP-CH_3I as dark solids.

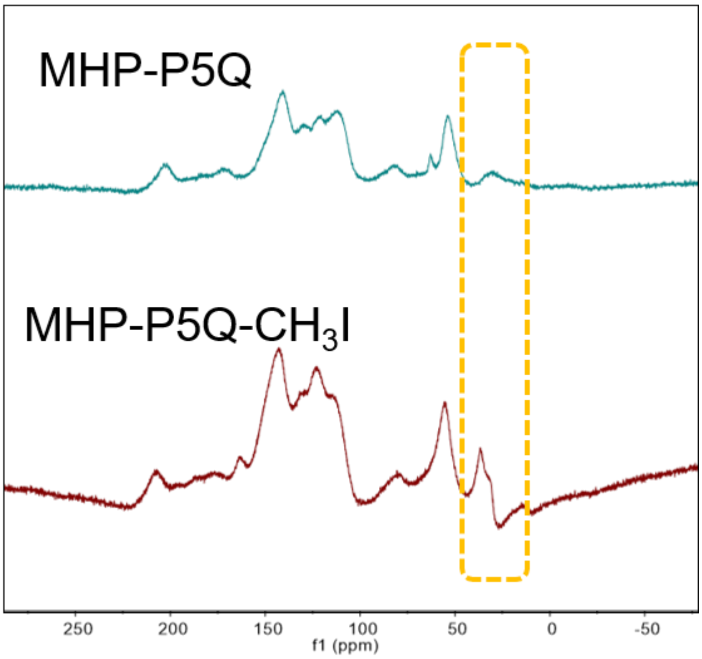

Supplementary Figure 32. ^{13}C CP-MAS NMR spectra of MHP-P5Q and MHP-P5Q-CH_3I.

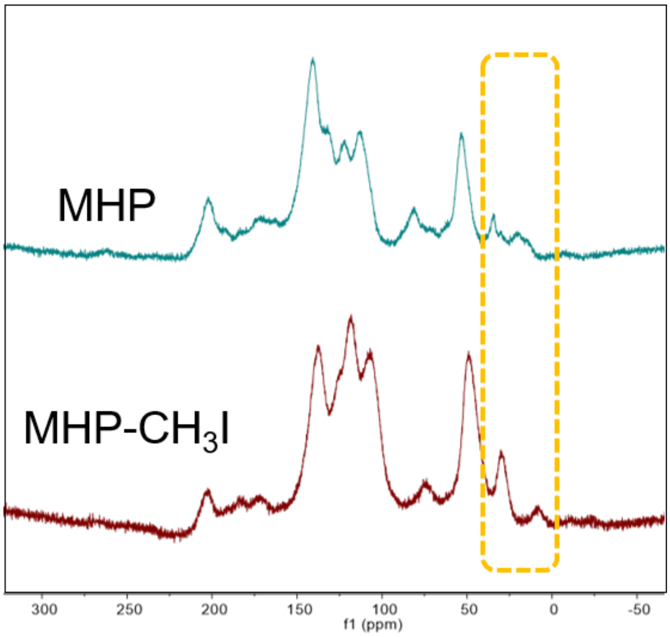

Supplementary Figure 33. ^{13}C CP-MAS NMR spectra of MHP and MHP-CH_3I.

6.4 Quantum Chemical Calculation Details

All the calculations are done with Gaussian 09 program package using the MP2 method.[S6] The LANL2DZ basis set with effective core potential (ECP)[S7] is employed for Iodine together with the 6-311G** basis set for the lighter elements H, C, O and N.[S8] Stationary points are found using the standard Berny optimization technique implemented in Gaussian 09 and confirmed via the vibrational analysis to make sure that minima show no imaginary frequency. After geometry optimization, the binding energy is estimated as $\Delta E_{binding}$ = E(complex) – E(dihydrophenazine) – E(CH_3I) + ΔE_{BSSE}, with ΔE_{BSSE} being the Basis Set Superposition Error (BSSE) correction.

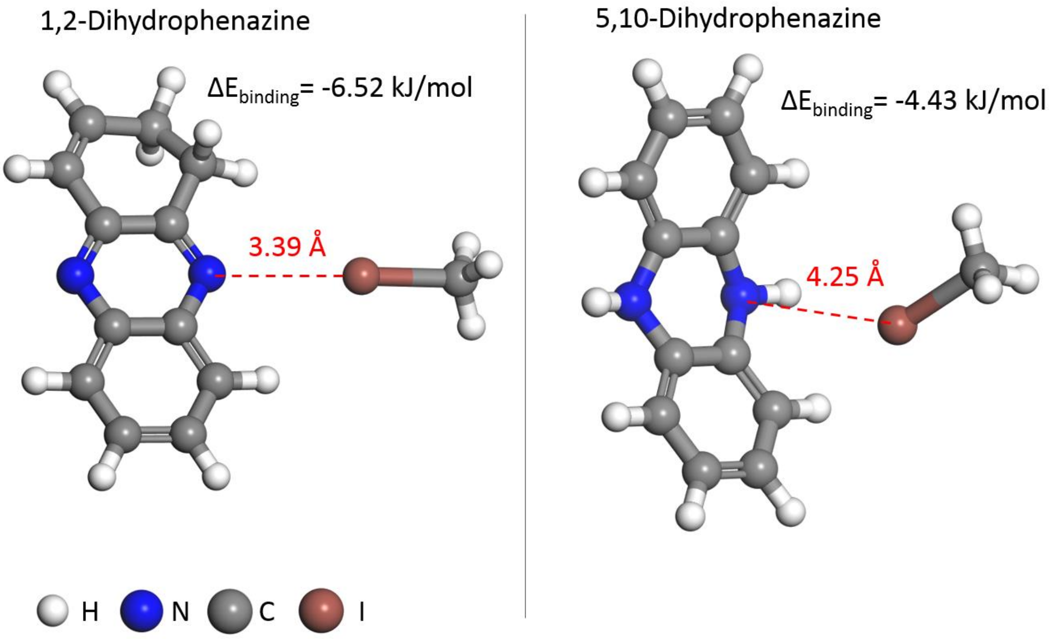

Supplementary Figure 34. Binding configuration of CH_3I with 1,2-Dihydrophenazine (left) and 5,10-Dihydrophenazine (right).

6.5 Crystal Structure Analysis

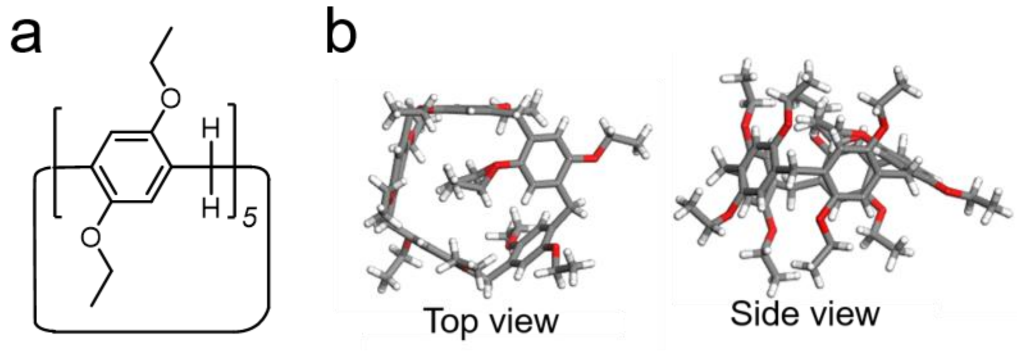

Supplementary Figure 35. (a) Chemical structure of EtP5 and (b) single-crystal x-ray structure of guest-free EtP5.

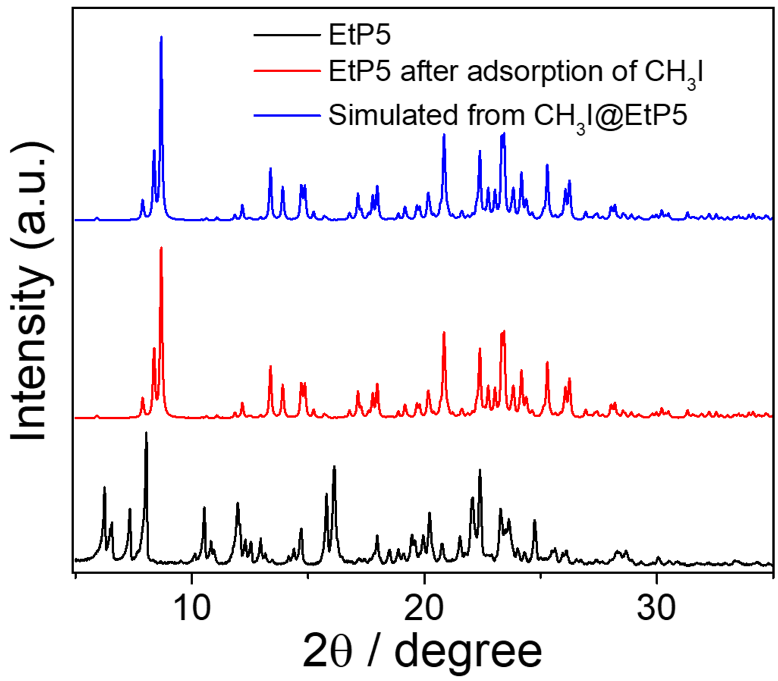

Supplementary Figure 36. PXRD patterns: EtP5, EtP5 after adsorption of CH_3I and simulated from single-crystal structure of CH_3I@EtP5.

References

S1. Mastalerz, M., Sieste, S., Ceni, M. & Oppel, I. M. Two-Step Synthesis of Hexaammonium Triptycene: An Air-Stable Building Block for Condensation Reactions to Extended Triptycene Derivatives. *J. Org. Chem.* **76**, 6389 (2011).

S2. Han, C., Zhang, Z., Yu, G. & Huang, F. Syntheses of A Pillar[4]arene[1]quinone and A Difunctionalized Pillar[5]arene by Partial Oxidation. *Chem. Commun.* **48**, 9876 (2012).

S3. Zhu, Z., Hong, M., Guo, D., Shi, J., Tao, Z. & Chen, J. All-Solid-State Lithium Organic Battery with Composite Polymer Electrolyte and Pillar[5]quinone Cathode. *J. Am. Chem. Soc.* **136**, 16461 (2014).

S4. Hu, X.-B., Chen, Z., Chen, L., Zhang, L., Hou, J.-L. & Li, Z.-T. Pillar[*n*]arenes (*n* = 8–10) with Two Cavities: Synthesis, Structures and Complexing Properties. *Chem. Commun.* **48**, 10999 (2012).

S5. Li, B., Dong, X., Wang, H., Ma, D., Tan, K., Jensen, S., Deibert, B. J., Butler, J., Cure, J., Shi, Z., Thonhauser, T., Chabal, Y. J., Han, Y. & Li, J. Capture of Organic Iodides from Nuclear Waste by Metal-Organic Framework-Based Molecular Traps. *Nat. Commun.* **8**, 485 (2017).

S6. M. J. Frisch, G. W. Trucks, H. B. Schlegel, G. E. Scuseria, M. A. Robb, J. R. Cheeseman, G. Scalmani, V. Barone, B. Mennucci, G. A. Petersson, H. Nakatsuji, M. Caricato, X. Li, H. P. Hratchian, A. F. Izmaylov, J. Bloino, G. Zheng, J. L. Sonnenberg, M. Hada, M. Ehara, K. Toyota, R. Fukuda, J. Hasegawa, M. Ishida, T. Nakajima, Y. Honda, O. Kitao, H. Nakai, T. Vreven, J. A. Montgomery Jr, J. E. Peralta, F. Ogliaro, M. Bearpark, J. J. Heyd, E. Brothers, K. N. Kudin, V. N. Staroverov, R. Kobayashi, J. Normand, K. Raghavachari, A. Rendell, J. C. Burant, S. S. Iyengar, J. Tomasi, M. Cossi, N. Rega, J. M. Millam, M. Klene, J. E. Knox, J. B. Cross, V. Bakken, C. Adamo, J. Jaramillo, R. Gomperts, R. E. Stratmann, O. Yazyev, A. J. Austin, R. Cammi, C. Pomelli, J. W. Ochterski, R. L. Martin, K. Morokuma, V. G. Zakrzewski, G. A. Voth, P. Salvador, J. J. Dannenberg, S. Dapprich, A. D. Daniels, Ö. Farkas, J. B. Foresman, J. V. Ortiz, J. Cioslowski, D. J. Fox, Gaussian, Inc., Wallingford CT, 2010

S7 Danovich, D.; Hrušák, J.; Shaik, S. Ab Initio Calculations for Small Iodo Clusters. Good Performance of Relativistic Effective Core Potentials. *Chem. Phys. Lett.* **233**, 249 (1995)

S8 Hariharan, P. C.; Pople, J. A., The Influence of Polarization Functions on Molecular Orbital Hydrogenation Energies. *Theor. Chim. Acta.* **28**, 213 (1973).
